# A Genomic Analysis of *Bacillus megaterium* HT517 Reveals the Genetic Basis of Its Abilities to Promote Growth and Control Disease in Greenhouse Tomato

**DOI:** 10.1155/2022/2093029

**Published:** 2022-12-27

**Authors:** Wei Yang, Yingnan Zhao, Yang Yang, Minshuo Zhang, Xiaoxi Mao, Yanjie Guo, Xiangyu Li, Bu Tao, Yongzhi Qi, Li Ma, Wenju Liu, Bowen Li, Hong J. Di

**Affiliations:** ^1^State Key Laboratory of North China Crop Improvement and Regulation, Key Laboratory for Farmland Eco-Environment of Hebei Province, Hebei Collaborative Innovation Center for Green & Efficient Vegetable Industry, College of Resources and Environmental Science, Hebei Industrial Technology Institute of Microbial Fertilizers, Hebei Agricultural University, Baoding 071000, China; ^2^College of Plant Protection, Hebei Agricultural University, Baoding 071000, China; ^3^Agricultural and Rural Bureau of Yongqing, Hebei Province 065600, China; ^4^Centre for Soil and Environmental Research, Lincoln University, Christchurch 7647, New Zealand

## Abstract

*Bacillus megaterium* is well known as a plant growth-promoting rhizobacterium, but the relevant molecular mechanisms remain unclear. This study aimed to elucidate the effects of *B. megaterium* HT517 on the growth and development of and the control of disease in greenhouse tomato and its mechanism of action. A pot experiment was conducted to determine the effect of *B. megaterium* on tomato growth, and this experiment included the HT517 group (3.2 × 10^8^ cfu/pot) and the control group (inoculated with the same amount of sterilized suspension). An antagonistic experiment and a plate confrontation experiment were conducted to study the antagonistic effect of *B. megaterium* and *Fusarium oxysporum f.*sp. *lycopersici*. Liquid chromatography–mass spectrometry was used to determine the metabolite composition and metabolic pathway of HT517. PacBio+Illumina HiSeq sequencing was utilized for map sequencing of the samples. An in-depth analysis of the functional genes related to the secretion of these substances by functional bacteria was conducted. HT517 could secrete organic acids that solubilize phosphorus, promote root growth, secrete auxin, which that promotes early flowering and fruiting, and alkaloids, which control disease, and reduce the incidence of crown rot by 51.0%. The complete genome sequence indicated that the strain comprised one circular chromosome with a length of 5,510,339 bp (including four plasmids in the genome), and the GC content accounted for 37.95%. Seven genes (*pyk*, *aceB*, *pyc*, *ackA*, *gltA*, *buk*, and *aroK*) related to phosphate solubilization, five genes (*trpA*, *trpB*, *trpS*, *TDO2*, and *idi*) related to growth promotion, eight genes (*hpaB*, *pheS*, *pheT*, *ileS*, *pepA*, *iucD*, *paaG*, and *kamA*) related to disease control, and one gene cluster of synthetic surfactin were identified in this research. The identification of molecular biological mechanisms for extracellular secretion by the HT517 strain clarified that its organic acids solubilized phosphorus, that auxin promoted growth, and that alkaloids controlled tomato diseases.

## 1. Introduction

In 1935, the former Soviet scholar Monkina isolated *Bacillus megaterium* var. phosphaticum (*B. megaterium*), which could decompose organophosphorus compounds, such as lecithin or nucleic acid from chernozem, applied it to decompose organophosphorus compounds in soil, and started research on the artificial production of phosphorus bacterial fertilizer [1]. In recent years, with the wide application of microbial fertilizers in agriculture, *B. megaterium* has been studied intensively for its phosphorus dissolving function in soil and has become a common microbial fertilizer strain, playing an exceptional role in dissolving phosphorus and potassium, promoting growth, and controlling plant diseases [2–5]. Although the absolute content of phosphorus in the soil was high, the availability of phosphorus was low, mainly in the form of insoluble inorganic phosphorus or organophosphorus, which was difficult for plants to directly absorb and utilize [6]. At present, it is widely believed that the dissolution mechanism of inorganic phosphorus mainly includes dissolving insoluble phosphate directly by secreting organic acids (lactic acid, succinic acid [7], gluconic acid, citric acid, and oxalic acid) and releasing phosphate through chelating metal ions in soil [8]. The primary mechanism of organophosphorus dissolution is enzymatic hydrolysis [9]. It was found in previous studies that the application of a microbial inoculant containing *B. megaterium* during the planting period could significantly increase the content of available P and available K in pepper rhizosphere soil by approximately 30% and the phosphorus and potassium uptake of pepper plants by approximately 40%, significantly reducing the incidence of soil-borne diseases [10]. Other studies showed that *B. megaterium* JD-2 had a strong ability to dissolve organophosphorus and inorganic phosphorus, and its fermentation broth could increase the available phosphorus content by two times after being inoculated into soil [11]. The soil available P concentration was significantly increased by 22.8%, 135%, and 95.1% by applying a microbial inoculant containing *B. megaterium* and *Paenibacillus mucilaginosus* in lightly salinized soil planted with tomato, watermelon, and melon [12].

Considering the mechanisms of *B. megaterium*, *B. megaterium* can promote cucumber yield by producing 3-indoleacetic acid, and it can produce cytokinin at low concentrations, promoting soybean plant growth [13]. *B. megaterium* JX285 secretes organic acids that solubilize phosphate and has a gene related to citrate synthase synthesis in its gene sequence [14]. Phosphate-solubilizing bacteria can secrete many extracellular enzymes, which play an active role in the mineralization of soil organophosphorus [15]. Some studies have shown that *B. megaterium* can produce two kinds of antagonistic factors, such as lipopeptide antibiotics and antagonistic proteins, which have antibacterial effects on pathogenic bacteria [16]. A surfactant secreted by *B. megaterium* had antiviral, antimycoplasma, and antibacterial activities and could destroy viral lipid membranes [17]. Surfactants are controlled by the *sfp* coding gene, and the ability of wild *B. megaterium* to produce surfactant is positively correlated with its ability to produce inducible resistant substances and its resistance to gray mold [18]. *B. megaterium* HGS7 consistently exhibits antagonistic activity against phytopathogens and strong tolerance to abiotic stress in vitro [19]. In recent studies, genomic analysis has become an effective segmental approach in exploring strain function [20–23]. In general, most studies on the growth promotion and disease control capabilities of *B. megaterium* have focused on effect testing. Although it has been revealed that *B. megaterium* secretes organic acids, auxin, and antimicrobials according to the literature [14, 16, 17], few studies have investigated the expression of related genes in *B. megaterium*. Complete genome sequencing would be more helpful to explain the mechanisms of *B. megaterium* biochemical activities, which would have high academic and practical value.

Based on the aforementioned, this study performed whole genome sequencing of *B. megaterium* (HT517) to explore related genes based on its effects of growth promotion and disease control. To achieve this aim, a greenhouse tomato pot experiment was conducted to verify the growth promotion and disease resistance effects of *B. megaterium*. The composition of the extracellular secretion was analyzed by liquid chromatography–mass spectrometry. PacBio+Illumina HiSeq whole-genome sequencing was utilized to analyze genes and metabolic pathways related to the effects of growth promotion and disease control. These analyses may help identify the functional genes of *B. megaterium* with the aim of enhancing the utilization of the positive biochemical capabilities of this strain.

## 2. Materials and Methods

### 2.1. Pot Experiment

#### 2.1.1. Suspension of Potted Bacteria

The bacterial strain used in this study was *B. megaterium* (HT517), provided by Hebei Runwo Biotechnology Company, Dongmagezhuang Village, Anci District, Langfang City, Hebei Province, China; the *B. megaterium* inoculant was prepared with an initial content of 1.6 × 10^8^ cfu/mL. A suspension of *Fusarium oxysporum f.*sp. *lycopersici* (Pathogen F) was provided by the College of Plant Protection of Hebei Agricultural University, Baoding, China, with an initial viable fungal number of 1.3 × 10^9^ cfu/mL.

#### 2.1.2. Experimental Design

The experimental design included two treatments. The first treatment group involved an experiment to identify the effects of *B. megaterium* on tomato growth, in which each pot containing a normal tomato seedling was inoculated with 2 mL of a suspension of *B. megaterium* (HT517) at the cell concentration mentioned above; as a control (CK), each pot/tomato seedling was inoculated with 2 mL of a sterilized suspension of *B. megaterium* (HT517) at the same cell concentration as the experimental group. The second treatment group was an antagonistic experiment to study the effects on tomato seedlings of a dual inoculation of the beneficial *B. megaterium* and the common tomato pathogen *F. oxysporum f.*sp. *lycopersici*, which we called pathogen F. Each pot containing a tomato seedling that had been infected with pathogen F as described below was inoculated with 2 mL of a suspension of *B. megaterium* (HT517), and as a control, tomato seedlings treated with sterilized pathogen F (following the same procedure as described below) were inoculated with 2 mL of sterilized *B. megaterium* (HT217) bacterial suspension. All treatment groups included three replicates.

According to the experimental scheme, tomato seedlings were planted in plastic pots with a diameter of 15 cm, and each pot was filled with 500 g of 2 mm sieved dry soil and one tomato plant. To observe and record, every 10 pots were a basic treatment unit, randomly arranged in the climate chamber. For the treatments of tomato seedlings infected by pathogen F, the tomato seedlings were washed with sterile water to remove root matrix before transplanting, and then, the epidermis of the stem base was scratched 1.0 cm × 0.5 cm with a sterilized scalpel. Tomato roots were immersed in pathogen F spore suspension (or sterilized Pathogen F suspension) for 5 minutes before transplanting. The *B. megaterium* treatments were inoculated with bacterial suspensions, and CK was inoculated with the same amount of sterilized bacterial suspension. After transplanting, 100 mL of water was irrigated in each pot, and then, 100 mL of water was irrigated every two days. The conventional management of tomato plants was consistent among the different treatments. Plant growth and disease were observed regularly after transplanting.

### 2.2. Sample Collection and Measurement

At 30 days after tomato transplanting, the plant height, stem diameter, root weight, leaf number, chlorophyll, and shoot biomass were measured. In addition, the flowering time of each treatment was observed and recorded from the time of transplanting to the time of first flowering, and the average value was taken as the early flowering time. The disease condition of each treatment was observed, and the number of diseased plants in different treatments was recorded from transplanting to the end of the experiments.

### 2.3. Plate Confrontation Experiment

A plate confrontation experiment was carried out to study the interactions of *B. megaterium* and the fungal pathogen F. A piece of pathogenic fungus mat was inoculated/placed in the center of a PDA(Potato Dextrose Agar) plate, and the *B. megaterium* (HT517) was inoculated onto the agar surface approximately 2 cm from the fungal inoculum using a sterilized toothpick. The bacteria/fungus co-culture was maintained at 28°C for 4 days, and the presence and size of the inhibition zones were observed and recorded. The experiment consisted of 2 treatments and 10 replicates. Treatment 1: inoculated with *B. megaterium* (HT517); treatment 2: no inoculation of *B. megaterium* as a blank control (CK). The calculation method of inhibition mycelial growth rate (%): (average diameter of control group − average diameter of treated group)/average diameter of control group × 100%.

### 2.4. Analysis and Determination of Extracellular Metabolites and Whole Genome Sequencing

First, the shake flask culture medium of *B. megaterium* HT517 was prepared. The formula was peptone 1.0%, yeast extract 0.7%, starch 2.0%, MgSO_4_ 0.2%, K_2_HPO_4_ 0.2%, (NH_4_)_2_SO_4_ 0.2%, and agar 2.0% (pH 7.2). The HT517 strain was then inoculated on sporogenous medium, activated at 28°C for 3 days, transferred to a shake flask, and cultured at 28°C for 12 hours until the log phase of growth. After centrifugation for 10 minutes at 10,000 rpm using a low-temperature (4°C) centrifuge, the supernatant was transferred to a sterile drying tube, frozen at −80°C, and freeze-dried to obtain *B. megaterium* HT517 freeze-dried powder, which was used for the analysis and determination of extracellular metabolites.

At the same time, the lower thallus was repeatedly washed and centrifuged with sterile water five times and then transferred to a sterile EP(eppendorf) tube and stored at −80°C for freezing to obtain the test sample of *B. megaterium* HT517. Shanghai Majorbio Technology Co., Ltd., Shanghai, China, conducted the whole genome sequencing analysis.

### 2.5. Statistical Analysis

Data analysis and visualization were performed using the IBM SPSS Statistics 26.0 and Excel 2019 software. The datasets involved in this study were subjected to Tukey's multiple comparison tests according to the least significant difference values at the level of *p* = 0.05, and independent-sample *T* tests with significance levels set to *p* = 0.05 or *p* = 0.01. The metabolomics and genomics data were analyzed using the Majorbio Cloud platform of Shanghai Majorbio Biopharm Technology Company, Shanghai, China.

## 3. Results

### 3.1. Effects of *B. megaterium* on Tomato Growth and Development

At 30 days after tomato transplanting, compared with CK, the root weight, plant height, stem diameter, leaf number, chlorophyll content, and shoot biomass in the HT517 treatment were significantly increased by 108.41%, 30.00%, 34.49%, 47.22%, 7.79%, and 31.23%, respectively. In addition, the early flowering time of tomato was 8.2 days earlier, and the number of fruit ears increased by 1.1 counts with the HT517 treatment (Table 1). These results indicated that *B. megaterium* HT517 significantly promoted the growth, early flowering time, and increased fruiting of tomato.

### 3.2. Effects of *B. megaterium* on Tomato; the Fungal Tomato Pathogen Fusarium

**Figure 1:**
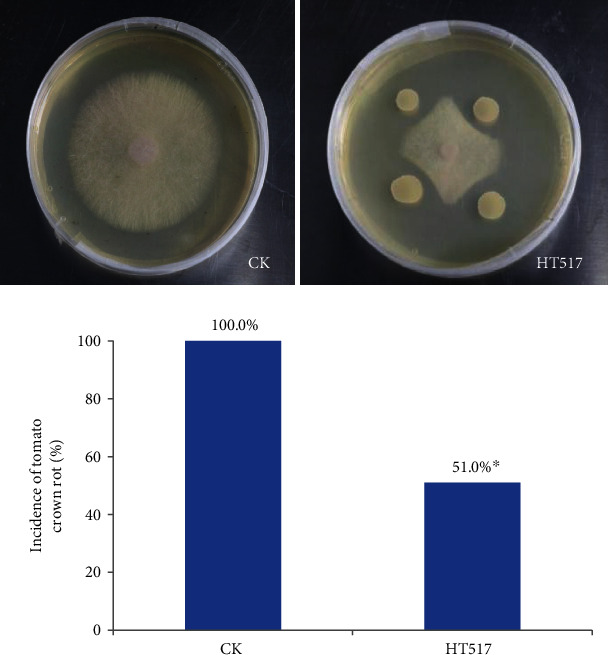
Effects of *Bacillus megaterium* on fungal tomato pathogen *Fusarium oxysporum f.*sp. *lycopersici*.

At 30 days after tomato transplanting, the effect of *B.megaterium* on tomato infected with fungal pathogen F was studied by inoculating tomato seedlings with *B. megaterium* HT517 and *F. oxysporum f.*sp. *lycopersici*. *B. megaterium* HT517 effectively inhibited the growth of *F. oxysporum f.*sp. *lycopersici* mycelium, and the inhibition rate was 57.26% (Figure 1). In terms of plant morbidity, there were significant differences between HT517 and CK.

The potted antagonistic experiment of tomato seedlings inoculated with *F. oxysporum f.*sp. *lycopersici* showed that *B. megaterium* HT517 significantly reduced the incidence of tomato crown rot by 51.0% (Figure 1), indicating that *B. megaterium* HT517 could effectively inhibit continuous cropping diseases.

### 3.3. Compositional Analysis of Extracellular Metabolites of *B. megaterium*

**Figure 2:**
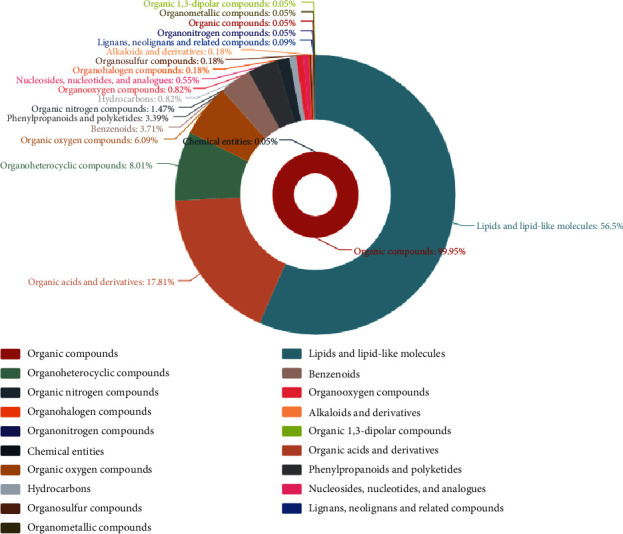
Classification of extracellular metabolites of *Bacillus megaterium.*

As shown in Figure 2, the fermentation centrifuge supernatant of *B. megaterium* HT517 could be divided into the following: lipids and lipid-like molecules accounted for 56.50%, organic acids and derivatives accounted for 17.18%, organoheterocyclic compounds accounted for 8.01%, organic oxygen compounds accounted for 6.09%, benzenoids accounted for 3.71%, phenylpropanoids and polyketides accounted for 3.39%, organic nitrogen compounds accounted for 1.47%, organooxygen compounds accounted for 0.82%, hydrocarbons accounted for 0.82%, nucleosides, nucleotides, and analogs accounted for 0.55%, organohalogen compounds accounted for 0.18%, alkaloids and derivatives accounted for 0.18%, lignans, neolignans, and related compounds accounted for 0.09%, organic compounds accounted for 0.05%, organonitrogen compounds accounted for 0.05%, organometallic compounds accounted for 0.05%, and organic 1,3-dipolar compounds accounted for 0.05%. Extracellular metabolites were mainly composed of lipids and lipid-like molecules, organic acids and derivatives, organoheterocyclic compounds, organic oxygen compounds, benzenoids, phenylpropanoids, and polyketides, accounting for 95.51% of the total components. Interestingly, some macromolecular compounds, including organic acids and derivatives (lipids, sugar, and benzene), activated soil nutrients and promoted the formation of soil clay.

### 3.4. Main Metabolic Pathways of *B. megaterium*

**Figure 3:**
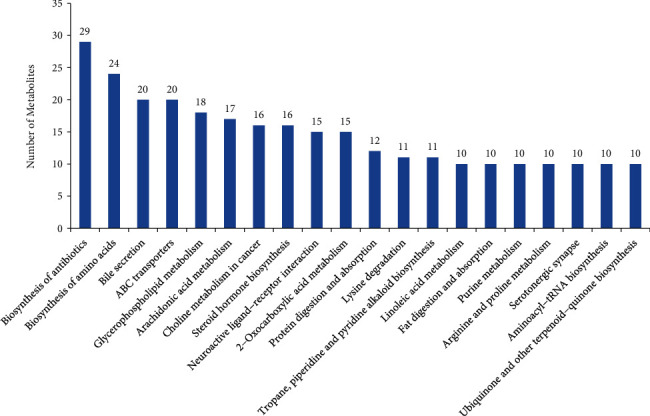
First 20 metabolic pathways in *Bacillus megaterium.*

The extracellular metabolites of *B. megaterium* HT517 were matched with the Kyoto Encyclopedia of Genes and Genomes (KEGG) database, and 1,204 substances participated in 251 metabolic pathways, including 3 conventional metabolic pathways and the number of substances involved: 161 metabolic pathways, 70 biosynthesis of secondary metabolites pathways, and 27 microbial metabolism pathways. As shown in Figure 3, the top 20 metabolic pathways and the number of substances involved in *B. megaterium* HT517 were as follows: 29 biosynthesis of antibiotics, 24 biosynthesis of amino acids, 20 bile secretion, 20 ATP-binding cassette transporters, 18 glycerophospholipid metabolism, 17 arachidonic acid metabolism, 16 choline metabolism in cancer, 15 neuroactive ligand–receptor interaction, 15 2-oxocarboxylic acid metabolism, 12 protein digestion and absorption, 11 lysine degradation, 11 tropane, piperidine, and pyridine alkaloid biosynthesis, 10 linoleic acid metabolism, 10 fat digestion and absorption, 10 purine metabolism, 10 arginine and proline metabolism, 10 serotonergic synapse, 10 aminoacyl-tRNA biosynthesis, and 10 ubiquinone and other terpenoid–quinone biosynthesis. Overall, the main metabolic pathways involved in *B. megaterium* HT517 included solubilization of phosphate and potassium, the hormones of flowering and fruiting, and antagonizing bacterial alkaloids.

**Figure 4:**
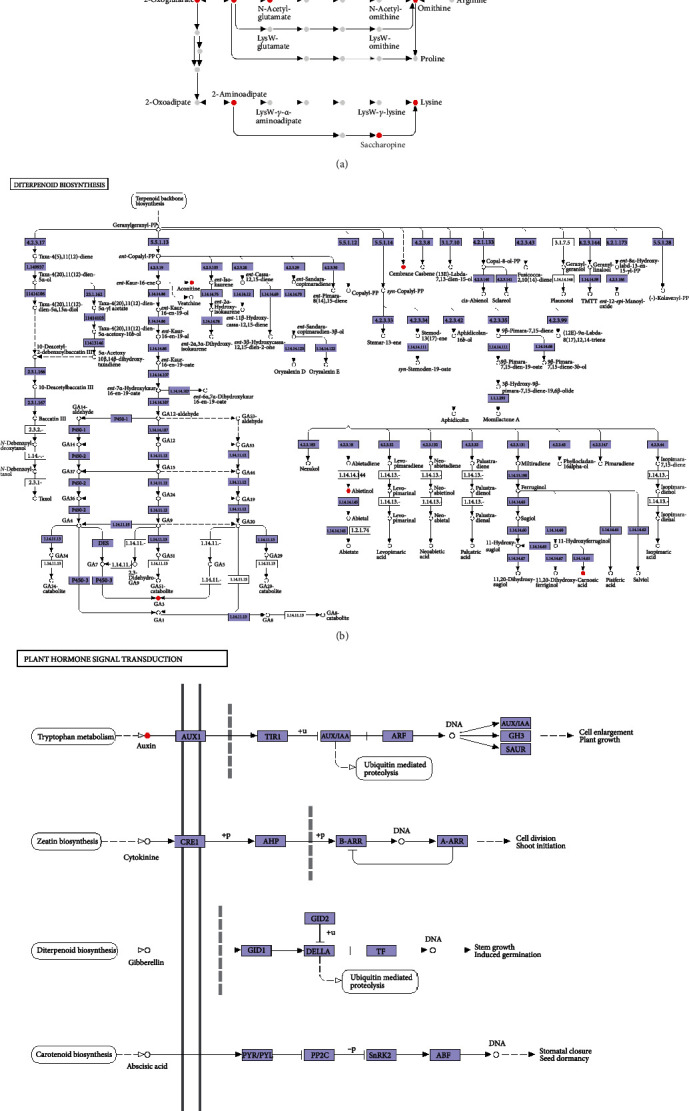
Metabolic pathways of *Bacillus megaterium*. (a) Amino acid biosynthesis. (b) Diterpenoid biosynthesis. (c) Plant hormone signal transduction.

**Figure 5:**
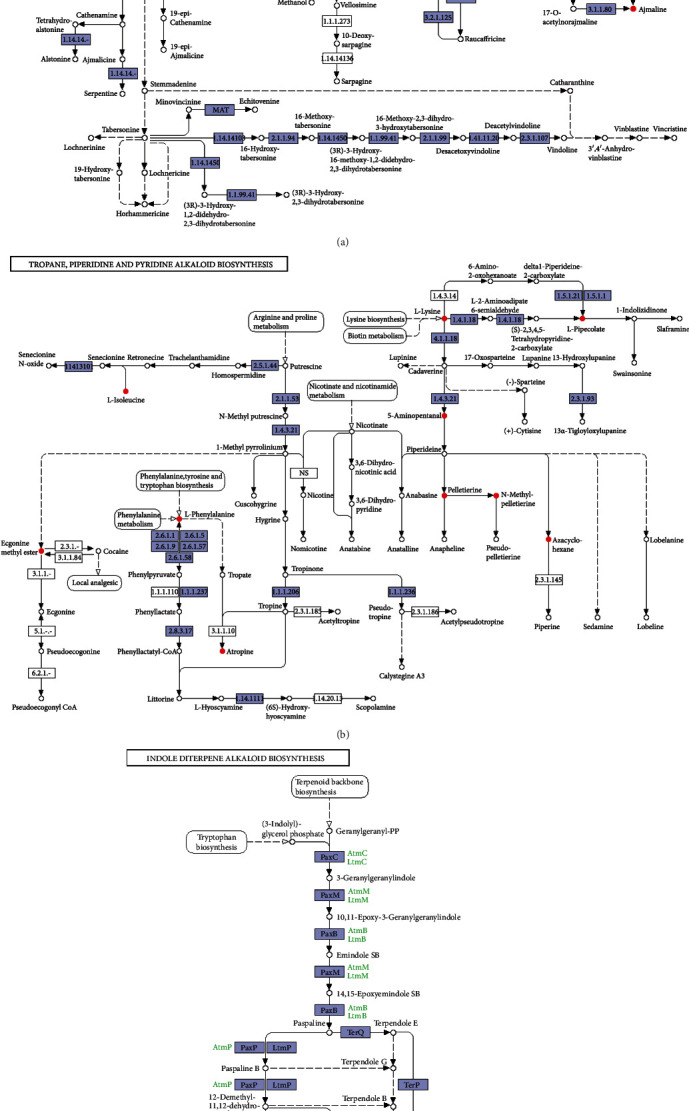
Metabolic pathways of *Bacillus megaterium*. (a) Biosynthesis of indole alkaloids. (b) Biosynthesis of tropane, pethidine, and pyridine alkaloids. (c) Indole diterpene alkaloid synthesis.

Figure 4 shows that *B. megaterium* HT517 could secrete 23 substances (Figure 4(a)), gibberellin A3 (Figure 4(b)), and 3-indoleacetic acid (Figure 4(c)) to participate in the biosynthesis of amino acids, diterpenoid biosynthesis, and plant hormone signal transduction. The red points in Figure 4 represent metabolites secreted by *B. megaterium* HT517 involved in the action sites of three metabolic pathways, ko01230, ko00904, and ko04075, indicating that *B. megaterium* HT517 could secrete amino acids, gibberellin A3, and 3-indoleacetic acid and stimulate cell enlargement by compounding AUX(Auxin)/IAA(Indoleacetic Acid), GH3(Indole 3 acetoamide synthetase), and SAUR(Auxin response protein), which could accelerate vegetative growth and reproductive growth, and increase root weight and early flowering and fruiting. As shown in Figure 5, *B. megaterium* HT517 could secrete ajmaline and tryptamine (Figure 5(a)), paxilline (Figure 5(c)), pelletierine, piperidine, ecgonine methyl ester, methylisopelletierine, phenyllactic acid, *L*-phenylalanine, *L*-lysine, *L*-isoleucine, *L*-pipecolic acid, 5-aminopentanal, and *D*-lysine (Figure 5(b)), which participated in the synthesis of indole alkaloid biosynthesis (Figure 5(a)), indole diterpene alkaloid biosynthesis (Figure 5(c)), and tropane, piperidine, and pyridine alkaloid biosynthesis (Figure 5(b)), respectively. The red points in Figure 5 show that the action sites of *B. megaterium* HT517 are involved in the three metabolic pathways of ko00901, ko00403, and ko00960. Therefore, *B. megaterium* HT517 could metabolize and synthesize alkaloids that had antagonistic effects on pathogenic bacteria, thus playing a role in preventing and controlling plant diseases.

### 3.5. Whole Genome of *B. megaterium*

**Figure 6:**
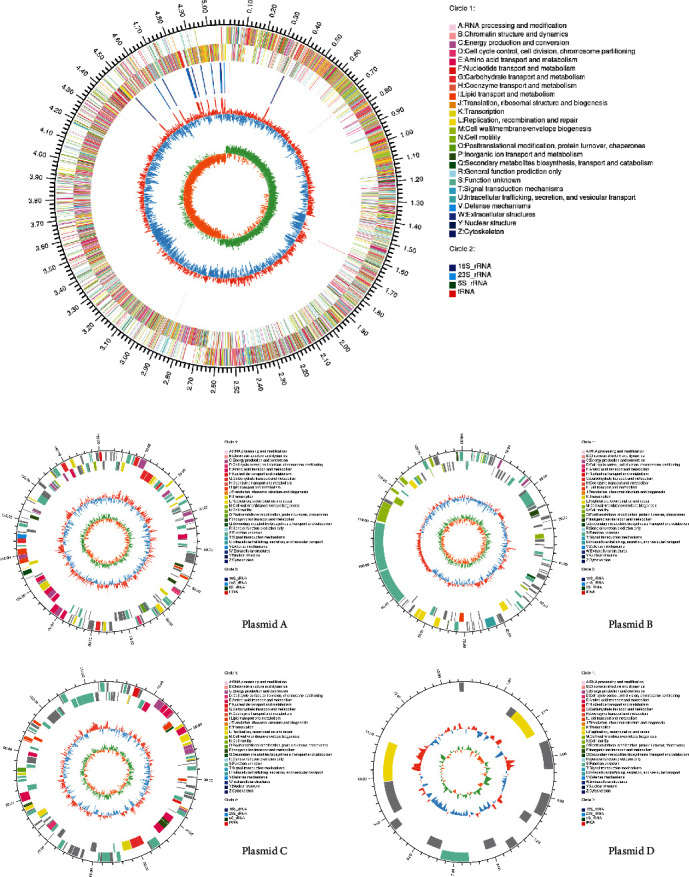
Circos evaluation of *Bacillus megaterium*.

As shown in Figure 6, the whole genome of *B. megaterium* HT517 was a ring chromosome with a length of 5,510,339 bp (including four plasmids in the genome), and GC(GC content is the proportion of Guanine and Cytosine in the whole genome of the subject) content accounted for 37.95%. The prediction showed that the genome had 5,923 CDSs(Coding sequences), and the coding sequence accounted for 82.10% of the whole chromosome sequence, with an average length of 763.76 bp. RNA gene analysis showed that the *B. megaterium* HT517 chromosome encoded 21 kinds of 116 tRNA genes and 40 rRNA genes, including 13 16S rRNA, 13 23S rRNA, and 14 5S rRNA. The prediction of repeat sequences showed that there were 90 repeated sequences, with a total length of 19,077 bp, accounting for 0.35% of the total chromosome length.

### 3.6. Prediction and Classification of Gene Function of *B. megaterium*

**Figure 7:**
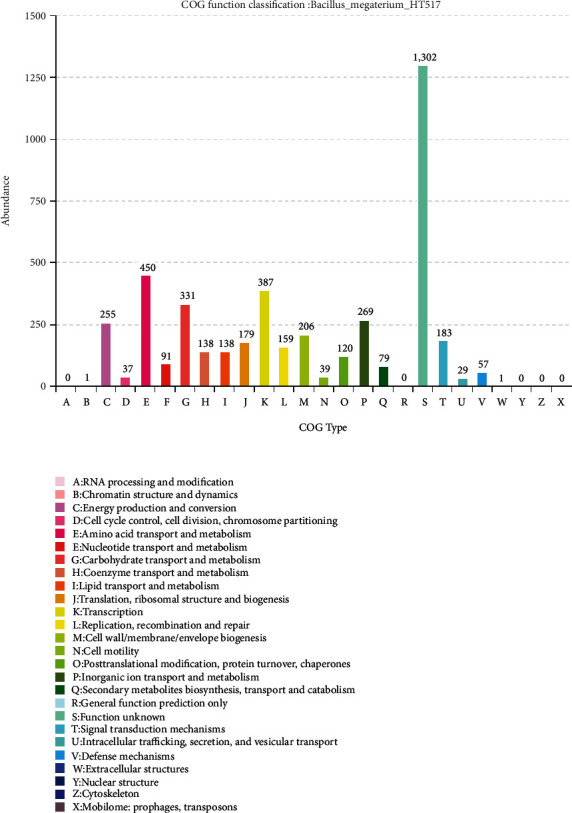
COG classification statistics of *Bacillus megaterium*.

The COG(Clusters of Orthologous Groups of proteins) functional classification genome of *B. megaterium* HT517 is shown in Figure 7. During the COG prediction process, 4,372 protein sequences were found to have COG numbers, accounting for 73.81% of the total predicted proteins. Relatively more chromosomal proteins were involved in information storage, processing, and metabolism, which accounted for 16.32% and 39.33% of the total predicted proteins, respectively. Among them, relatively more chromosomal protein sequences were involved in metabolism, transcription, and energy production and conversion, such as amino acid transport and metabolism, COG category K (transcription), COG category G (carbohydrate transport and metabolism), COG category P (inorganic ion transport and metabolism), and COG category C (energy production and conversion), accounting for 10.07%, 8.70%, 7.44%, 6.02%, and 5.75% of the total predicted proteins, respectively. It was summarized that *B. megaterium* HT517 had strong functions of secreting organic acids and transporting inorganic ions.

**Figure 8:**
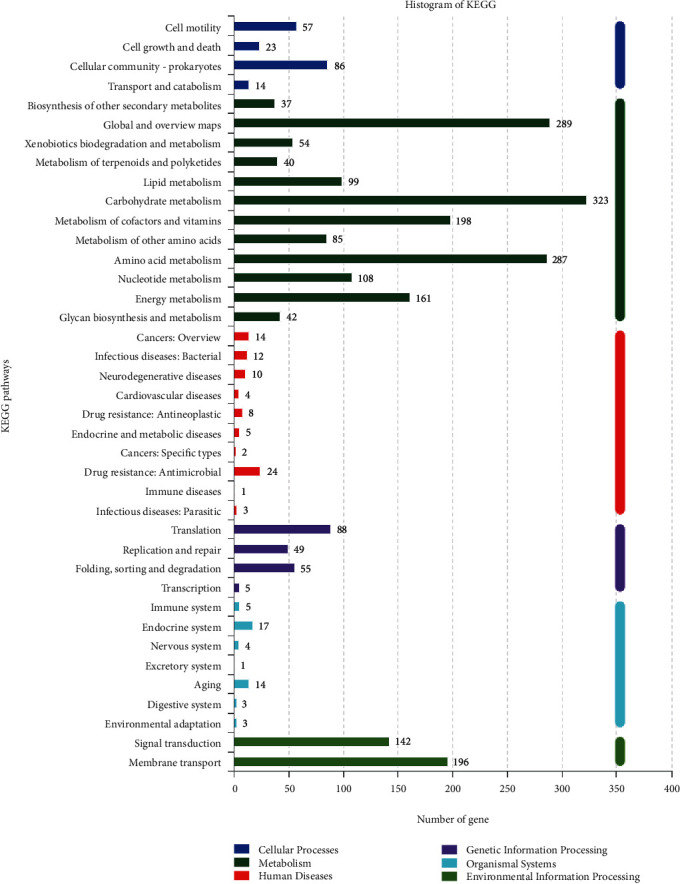
KEGG classification of *Bacillus megaterium*.

The KEGG database was used to analyze the metabolic pathways and regulatory networks of all genes predicted by the genome of *B. megaterium* HT517 (Figure 8). A total of 2,621 genes in the genome of *B. megaterium* HT517 were involved in 6 primary classified metabolic pathways and 39 secondary classified metabolic pathways, of which the primary classified metabolic pathways were present: cellular processes, metabolism, human diseases, genetic information processing, organismal systems, and environmental information processing. In the secondary classified metabolic pathway, the genome of *B. megaterium* HT517 was involved in 323 carbohydrate metabolism, 288 global and overview maps, 288 amino acid metabolism, 197 metabolism of cofactors and vitamins, 196 membrane transport, 160 energy metabolism, 142 signal transduction, 108 nucleotide metabolism, 99 lipid metabolism, and 88 translation.

### 3.7. Genes Related to Secretion of Organic Acids in Solubilizing Phosphorus by *B. megaterium*

According to the whole genome sequencing results of *B. megaterium* HT517 (Table 2), the functional genes and metabolic pathways were selected. For example, pyruvate, malate, citrate, shikimic, acetate, butyrate, and other organic acids, which had seven related functional genes *pyk*, *aceB*, *pyc*, *ackA*, *gltA*, *buk*, and *aroK*, were involved in the citric acid cycle, pyruvate metabolism, propionic acid metabolism, methyl butyrate metabolism, and the biosynthesis of phenylalanine, tyrosine, and tryptophan of 15 metabolic pathways of ko00020, ko00230, ko00400, ko00430, ko00620, ko00630, ko00640, ko00650, ko00680, ko00720, ko01200, ko01210, ko01230, ko04922, and ko05230. *B. megaterium* HT517 had the above 7 related functional genes and 15 metabolic pathways.

### 3.8. Genes Related to the Secretion of Auxin by *B. megaterium*

Five related functional genes of *trpA*, *trpB*, *trpS*, *TDO2*, and *idi* involved in the biosynthesis of terpenoid compounds, such as 3-indoleacetic acid and gibberellin were identified from the metabolic pathway of *B. megaterium* HT517, which is involved in the metabolism pathways of tryptophan, glycine, serine, threonine, phenylalanine, tyrosine, and terpenoid biosynthesis, namely ko00260, ko00380, ko00400, ko00900, ko00970, and ko01230 (Table 3). The results indicated that *B. megaterium* HT517 could stimulate vegetative growth and reproductive growth by participating in the biosynthesis of terpenoids, such as 3-indoleacetic acid and gibberellin, thus promoting root development and early flowering and fruiting.

### 3.9. Genes Related to the Secretion of Pathogenic Bacteria Antagonized by *B. megaterium*

**Figure 9:**
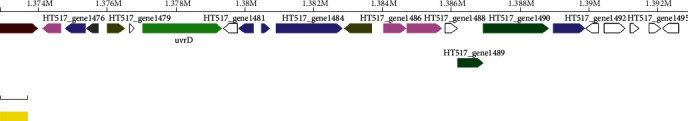
*Bacillus megaterium* surfactin biosynthetic gene cluster.

The KEGG database was used to analyze the metabolic pathways of *B. megaterium* HT517. It was found that eight related genes of *hpaB*, *pheS*, *pheT*, *ileS*, *pepA*, *iucD*, *paaG*, and *kamA* participated in the biosynthesis of scopolamine, pethidine, and pyridine alkaloids, which are involved in six metabolic pathways of ko00220, ko00310, ko00350, ko00360, ko00480, and ko00970 in the biosynthesis of amino acids, such as arginine, lysine, and tyrosine and the degradation of aromatic compounds, respectively (Table 4). It was indicated that *B. megaterium* HT517 could antagonize pathogens and prevent and control diseases by participating in the biosynthesis of scopolamine, pethidine, and pyridine alkaloids. Furthermore, by analyzing the secondary metabolite synthesis gene cluster of *B. megaterium* HT517 through the antiSMASH software, it was found that *B. megaterium* HT517 had a gene cluster consisting of 24 genomes that could synthesize bacteriostatic surfactin (Figure 9). Therefore, this could be the key factor of *B. megaterium* HT517 antagonizing *F. oxysporum f.*sp. *lycopersici*.

## 4. Discussion

In this experiment, it was obvious that *B. megaterium* promoted tomato roots and increased the quantity of roots by more than 108.4% (Table 1). We also found that *B. megaterium* could promote tomato flowering 8.2 days earlier and increase the average fruit ears by 1.1. The common natural auxin secreted is indole-3-acetic acid, which can promote the growth and morphological development of the root system and enhance the uptake of soil nutrients by plants [24]. Therefore, the growth of the plant root system can be promoted by secreting indoleacetic acid and cytokinin. Some studies have shown that hormones can stimulate early flowering and fruiting [25, 26]. Plant flowering is controlled by two hormones, “Florigen” and “Gibberellin.” When the level of “Florigen” is sufficient to stimulate the apical buds to blossom, the plants start to blossom, and the newly formed nutrient buds are temporarily inhibited by the local “anti-Florigen” signal [27]. Therefore, HT517 can produce gibberellin and other hormones to promote early flowering and fruiting of tomato.

We found that *B. megaterium* had a significant antagonistic effect against *F. oxysporum f.*sp. *lycopersici*, and inoculation with *B. megaterium* suspension could significantly reduce the incidence of tomato crown rot by more than 50%. Previous studies have also found that *B. megaterium* has an antagonistic function against pathogens [16, 28–30]. *B. megaterium* can inhibit the growth of pathogenic bacteria, such as tomato bacterial wilt and watermelon fusarium wilt [31]. *B. megaterium* can inhibit aflatoxin biosynthesis in peanuts through competitive growth and the production of secondary metabolites [32]. Unsaturated fatty acids produced by *B. megaterium* L2 could inhibit the growth of filamentous fungi and yeasts [33]. Therefore, the antagonistic effect of *B. megaterium* on *F. oxysporum f.sp. lycopersici* may be due to the production of lipopeptide secondary metabolites, although this will require further study.

Through the complete genome mapping analysis of *B. megaterium* HT517, we found that *B. megaterium* HT517 had seven functional genes (*pyk*, *aceB*, *pyc*, *ackA*, *gltA*, *buk*, and *aroK*) related to the secretion of organic acids. This was consistent with seven functional genes related to phosphorus solubilization identified in the study of *Bacillus mycoides* Gnyt1 [34]. It was confirmed that phosphorus-solubilizing bacteria dissolved insoluble phosphorus sources mainly by secreting small molecular organic acids, such as oxalic acid, succinoplatinic acid, lactic acid, citric acid, fumaric acid, and malic acid [35]. Phosphorus-solubilizing microorganisms can secrete many organic acids, such as citric acid, oxalic acid, tartaric acid, malic acid, and lactic acid, thus reducing soil pH [36]. Whole-genome sequence analysis of *B. megaterium* JX285 showed that it had functional genes, such as citrate synthase related to organic acid synthesis [14]), and the mechanism of its secretion of organic acids to dissolve phosphorus was revealed.

From the metabolic pathways of the *B. megaterium* HT517 strain, five related functional genes (*trpA*, *trpB*, *trpS*, *TDO2*, and *idi*) involved in the biosynthesis of terpenoids, such as 3-indoleacetic acid and gibberellin, were found, and six metabolic pathways involved in metabolism and terpenoid biosynthesis, such as tryptophan, glycine, serine, threonine, phenylalanine, and tyrosine, were identified. There are five metabolic pathways involved in bacterial synthesis of 3-indoleacetic acid, including IAM(Indole acetamide), IPyA(Indole-3-pyruvate), TSO(Tryptophan side chain oxidase), TAM(Tryptamine), and IAN(Indole acetonitrile) [37]. Rhizosphere growth-promoting bacteria secrete plant hormones such as abscisic acid, auxin, gibberellin, and cytokinin, and promote plant growth and development by regulating nutrient source/sink conversion and coordinating nutrient balance [38]. Rhizosphere growth-promoting bacteria can regulate root development and growth by secreting plant hormones, such as 3-indoleacetic acid, gibberellin, cytokinin, secondary metabolites, and enzymes [39]. Therefore, the HT517 strain could stimulate the vegetative growth and reproductive growth of greenhouse tomato by participating in the synthesis of terpenoids, such as 3-indoleacetic acid and gibberellin, and we identified the mechanism of the HT517 strain in promoting root development and early flowering and fruiting.

Eight functional genes were found to be involved in the biosynthesis of hyosolamine, pethidine, and pyridine alkaloids, and a gene cluster consisted of 24 genomes from the metabolic pathway of the HT517 strain. The fundamental reason why *B. megaterium* HT517 antagonizes *F. oxysporum f.*sp. *lycopersici* was identified. It has been proven that *B. megaterium* can produce two antagonistic factors, lipopeptide antibiotics and antagonistic proteins, which have antimicrobial effects on pathogenic fungi [40]. The secreted surfactin has antiviral, anti-mycoplasma, and antibacterial activities, and could destroy viral lipid membranes [17]. Surfactin is controlled by the coding gene *sfp* of (Phosphopanthenyl mercaptoethylamine transferase) PPTase. The ability of wild-type *Bacillus* to produce surfactin was positively correlated with its ability to produce inducible resistant substances and its resistance to gray mold [18]. Alkaloids, such as scopolamine, exert an obvious control effect on tomato root knot nematodes [41]. Therefore, the HT517 strain could antagonize and control pathogenic fungi by participating in the biosynthesis of hyosolamine, pethidine, and pyridine alkaloids, thus revealing the mechanism of its antagonistic effect.

There are limitations in this study. For example, the experiment was a qualitative study with a small sample size and was a laboratory study. The analyses could not identify the quantitative determination of functional genes. With the further development of molecular biology technology, we will be able to study functional genes quantitatively.

## 5. Conclusion

The HT517 strain could secrete organic acids, such as pyruvate, malic acid, citric acid, shikimic acid, acetic acid, butyric acid, and other organic acids through metabolic pathways involved in the biosynthesis of diterpenoids, scopolamine, pethidine, and pyridine alkaloids, and had seven related functional genes: *pyk*, *aceB*, *pyc*, *ackA*, *gltA*, *buk*, and *aroK*. The HT517 strain secreted extracellular metabolites, such as 3-indoleacetic acid and gibberellin GA3, which accelerate the vegetative growth and reproductive growth of tomato, and five related functional genes were identified: *trpA*, *trpB*, *trpS*, *TDO2*, and *idi*. Moreover, the HT517 strain secreted antagonistic alkaloids, such as scopolamine, pethidine, and pyridine, and macromolecular organic compounds, involving eight related functional genes, such as *hpaB*, *pheS*, *pheT*, *ileS*, *pepA*, *iucD*, *paaG*, and *kamA*, and a gene cluster synthesizing surfactin, which may play a decisive role in antagonizing tomato pathogens.

## Figures and Tables

**Table 1:** Effects of *Bacillus megaterium* on tomato growth and development.

Treatments	Root biomass fresh weight (FW) (g)	Plant height (cm)	Stem diameter (mm)	Leaf number (piece)	Chlorophyll (mg/g)	Shoot biomass FW (g)	Flowering time (day)	Fruit setting (count)
Control (CK)	2.26 ± 0.05	21.00 ± 0.20	4.91 ± 0.03	84.00 ± 2.00	63.37 ± 1.20	11.27 ± 0.02	31.50 ± 0.34	1.20 ± 0.13
HT517	4.71 ± 0.01∗∗	27.30 ± 0.38∗∗	6.60 ± 0.08∗∗	123.67 ± 1.76∗∗	68.30 ± 1.02∗	14.79 ± 0.33∗∗	23.30 ± 0.26∗∗	2.20 ± 0.13∗∗

**Table 2:** Functional genes associated with the secretion of organic acids in solubilizing phosphorus by *Bacillus megaterium* and their metabolic pathways.

Functional gene	Function	KO ID	Pathway ID
*Pyk*	Pyruvate kinase	K00873	ko00230, ko00620, ko01200, ko01230, ko04922, and ko05230
*aceB*	Malate synthase	K01638	ko00620, ko00630, and ko01200
*Pyc*	Pyruvate carboxylase	K01958	ko00020, ko00620, ko00720, ko01200, and ko01230
*ackA*	Acetate kinase	K00925	ko00430, ko00620, ko00640, ko00680, ko00720, and ko01200
*gltA*	Citrate synthase	K01647	ko00020, ko00630, ko01200, ko01210, and ko01230
*Buk*	Butyrate kinase	K00929	ko00650
*aroK*	Shikimate kinase	K00891	ko00400 and ko01230

**Table 3:** Functional genes and metabolic pathways related to auxin synthesis in *Bacillus megaterium.*

Functional gene	Function	KO ID	Pathway ID
*trpA*	Tryptophan synthase alpha chain	K01695	ko00260, ko00400, and ko01230
*trpB*	Tryptophan synthase beta chain	K01696	ko00260, ko00400, and ko01230
*trpS*	Tryptophanyl-tRNA synthetase	K01867	ko00970
*TDO2*	Tryptophan 2,3-dioxygenase	K00453	ko00380
*idi*	Isopentenyl-diphosphate delta-isomerase	K01823	ko00900

**Table 4:** Functional genes and metabolic pathways of alkaloid secreted by *Bacillus megaterium.*

Functional gene	Function	KO ID	Pathway ID
*hpaB*	4-Hydroxyphenylacetate 3-monooxygenase	K00483	ko00350 and ko01220
*pheS*	Phenylalanyl-tRNA synthetase alpha chain	K01889	ko00970
*pheT*	Phenylalanyl-tRNA synthetase beta chain	K01890	ko00970
*ileS*	Isoleucyl-tRNA synthetase	K01870	ko00970
*pepA*	Leucyl aminopeptidase	K01255	ko00480
*iucD*	Lysine n6-hydroxylase	K03897	ko00310
*paaG*	2-(1,2-Epoxy-1,2-dihydrophenyl)acetyl-CoA isomerase	K15866	ko00360
*kamA*	Lysine 2,3-aminomutase	K01843	ko00310

## Data Availability

The data analyzed in this study are available from the first author upon reasonable request.
